# Epidemiology of *Chlamydia* sp. infection in farmed Siamese crocodiles (*Crocodylus siamensis*) in Thailand

**DOI:** 10.1186/s13028-023-00713-x

**Published:** 2023-11-27

**Authors:** Nae Tanpradit, Metawee Thongdee, Ladawan Sariya, Weena Paungpin, Somjit Chaiwattanarungruengpaisan, Wanna Sirimanapong, Tanit Kasantikul, Rassameepen Phonarknguen, Apichart Punchukrang, Paisin Lekcharoen, Nlin Arya

**Affiliations:** 1 Department of Clinical Sciences and Public Health, Faculty of Veterinary Science, Mahidol University, Nakhon Pathom, 73170 Thailand; 2 Monitoring and Surveillance Center for Zoonotic Diseases in Wildlife and Exotic Animals, Faculty of Veterinary Science, Mahidol University, Nakhon Pathom, 73170 Thailand; 3 Faculty of Veterinary Science, The Veterinary Aquatic Animal Research Health Care Unit, Mahidol University, Nakhon Pathom, 73170 Thailand; 4 Veterinary Diagnostic Laboratory, Michigan State University, East Lansing, USA; 5 Faculty of Agricultural Technology, Songkhla Rajabhat University, Songkhla, 90000 Thailand; 6 The Department of Veterinary Public Health, Faculty of Veterinary Science, Chulalongkorn University, Bangkok, 10330 Thailand; 7 Department of Pre-Clinic and Applied Animal Science, Faculty of Veterinary Science, Mahidol University, Nakhon Pathom, 73170 Thailand

**Keywords:** Chlamydiosis, Risk factors, Polymerase chain reaction, *Omp*A

## Abstract

**Background:**

Although *Chlamydia* sp. causes widespread disease outbreaks in juvenile crocodiles in Thailand, data regarding the epidemiology, and risk factors of such infections are limited. The aim of this study was to investigate the prevalence and possible risk factors associated with *Chlamydia* sp. infections on Siamese crocodile (*Crocodylus siamensis*) farms in Thailand. A cross-sectional study was conducted from July to December 2019. Samples were collected from 40 farms across six regions in Thailand. Conjunctival, pharyngeal, and cloacal swab samples were analyzed for *Chlamydiaceae* nucleic acids using semi-nested PCR followed by phylogenetic analysis based on the *omp*A gene fragment. Risk factors of infection were analyzed using chi-square and univariate regression to calculate odds ratios.

**Results:**

The prevalence of *Chlamydia* sp. infection across all regions was 65%. The *omp*A phylogenetic analysis showed that *Chlamydia* sp*.* detected in this study was genetically closely related to *Chlamydia crocodili* and* Chlamydia caviae.* The risk factors for infection were water source, reusing treated wastewater from the treatment pond, not disposing of leftover food, low frequency of water replacement in the enclosure of juvenile crocodiles, and lack of water replacement after the death of a crocodile.

**Conclusion:**

The prevalence of *Chlamydia* sp. infection in farmed crocodiles in Thailand was 65% during the study period. Cloacal swabs were superior to conjunctival and pharyngeal swabs due to their higher sensitivity in detecting *Chlamydia* sp., as well as their lower invasiveness. Good management and biosecurity in crocodile farming can reduce the risk of *Chlamydia* sp. infection.

**Supplementary Information:**

The online version contains supplementary material available at 10.1186/s13028-023-00713-x.

## Background

Crocodile farms in Thailand are very famous sites for the national tourism industry and well-known leather providers for international fashion products. There are over 1.2 million Siamese crocodiles (*Crocodylus siamensis)* across 1415 registered farms, based on the database of the Fisheries Department from 2021 [1]. However, an outbreak of *Chlamydia* sp. infection caused economic devastation and the loss of thousands of young crocodiles in 2012–2013, after which the infection became an endemic in Thailand [2, 3].

*Chlamydia* sp. is a Gram-negative obligate intracellular bacterium characterized by a unique biphasic developmental cycle that can infect a wide range of hosts, including Siamese crocodiles [4–6]. A survey on its molecular diversity revealed very high diversity, wide distribution, and high abundance of *Chlamydia* sp. in the hosts and the environment settings [7]. Infected crocodiles can either be asymptomatic or show nonspecific clinical signs, e.g., conjunctivitis, pharyngitis, ascites, depression, anorexia, death [5, 8, 9], kyphoscoliosis, and stunted growth. Diagnoses are based on gross and histopathological examination and molecular testing for 16S/23S rRNA and major outer membrane protein (*omp*A) genes. A new species, *Chlamydia crocodili*, was reported in 2021 [10]. Although chlamydiosis causes high mortality in juvenile crocodiles, our knowledge regarding its pathogenesis and risk factors is very limited. Crocodile farming management and hygiene in Thailand is diverse, and certain animal husbandry activities can pose risks for *Chlamydia* sp. infections. This study aims to investigate the epidemiology and potential risk factors associated with *Chlamydia* sp. infections on Siamese crocodile farms in Thailand.

## Methods

### Study design and sample collection

This cross-sectional epidemiological study was conducted between July and December 2019. Crocodile farms were selected from the list from the Department of Fisheries, Thailand according to the consent of the farm owners and the availability of the farm during the sample collection period. The number of farms from each region (North, Central, East, Northeast, West, and South) and the sample size from each farm were calculated with a 90% level of confidence, 10% precision, and an assumed 25% prevalence for *Chlamydia* sp. infection. As a result, samples were collected from 486 live crocodiles from 40 farms across six regions in Thailand. Crocodiles were randomly chosen within the farm for sample collection. Swab samples were collected using sterile rayon swabs (Puritan Medical Products Company, ME, USA) from three sites: the conjunctiva, pharynx, and cloaca. All samples were transported in transport media (sucrose/phosphate/glutamate buffer containing 500 μg/mL streptomycin, 500 μg/mL vancomycin, 50 μg/mL gentamycin, and 2.5 μg/mL fungizone) at 4 ℃ and stored at − 80 ℃ until DNA extraction.

### Risk factor analyses

Information about the type of pen, previous crocodile health status, and farm management practices was collected by interviewing farm practitioners. The 26 risk factors collected were as follows: the primary farm objectives, crocodile sources, crocodile species, presence of aquatic and avian livestock on the farm, presence of nearby livestock, feed source, food storage, feed additives, management of leftover feed, pen floor type, presence of shade, ratio of dry and wet area, pond preparation, pond cleaning practice, water source, water reservoir, reuse of treated wastewater from the treatment pond, routine water quality checking practice, frequency of water replacement for juveniles, water replacement after discovering a dead crocodile, dead crocodile management, routine health monitoring, previous health problems, signs of depression, and vertebral deformities. The data from each factor were categorized and underwent further statistical analysis of infection risks.

Univariate logistic regression was performed to calculate the odds ratios and 95% confidence intervals for the risk factor analyses between different factors and infection. The Chi-square test was used to determine P-values. Statistical significance was set at P < 0.05. All tests were conducted in IBM SPSS Statistics for Windows Ver. 25 (Statistical Package for the Social Sciences, IBM Corp., Armonk, NY, USA).

### Molecular detection of Chlamydiaceae

Genomic DNA was extracted from conjunctival, pharyngeal, and cloacal swabs using the Genomic DNA Mini Kit (Geneaid, New Taipei City, Taiwan). The extracted DNA was suspended in 30 µL of Tris–EDTA buffer and stored at −  20 ℃ until analysis. Semi-nested PCR using primers specific to the *omp*A gene was performed using a previously published method [11]. Briefly, primers A and B were used in the first round of PCR, and primers B, and C were used in the second round of PCR. In the first round, 25 μL of the PCR mixture contained 2 µL of template DNA, 2.5 µL of 10 × Mg^2+^-free buffer, 1.5 mM of Mg^2+^ solution, 1 mM of dNTPs, 2.5 units of i-*Taq* DNA polymerase (iNtRON Biotechnology, Inc., South Korea), and 0.5 µM of each primer. The cycling parameters were as follows: 2 min at 94 °C for initial denaturing, followed by 35 cycles of 30 s at 94 °C, 30 s at 58 °C, and 30 s at 72 °C, and termination at 72 °C for 7 min. After the first round of PCR, the second round of PCR was performed using primers B and C and the product from the first round as a template. The PCR mixture and PCR parameters were the same as those of the first round of PCR, except the use of Mg^2+^ at a final concentration of 3 mM. The second round of PCR generated a product 165 bp in size. A positive control (recombinant plasmid DNAs harboring the *omp*A gene fragment), negative control (nuclease-free water), and extraction of negative control (phosphate-buffered saline) were included in each PCR run.

### Phylogenetic marker amplification, DNA sequencing, and DNA analysis

Two *Chlamydia*-positive samples from each region (in total 12 samples from 6 regions) were randomly selected for *omp*A gene sequencing. A 1058 bp fragment of the *omp*A gene was amplified with primers CTU (5′ ATGAAA AAA CTC TTG AAA TCG G 3′) and CTL (5′ CAA GAT TTT CTA GAY TTC ATYTTG TT 3′) [12]. Briefly, 25 μL of PCR reaction mixture contained 2 μL of template DNA, 2.5 μL of 10 × PCR buffer containing 1.5 mM Mg^2+^, 1 mM dNTPs mix, 0.5 μL of i-*Taq* DNA polymerase (iNtRON Biotechnology, Inc., South Korea), and 0.5 μM each of forward and reverse primer. PCR was performed under the conditions of 2 min at 94 °C for initial denaturing, followed by 35 cycles of 30 s at 94 °C, 30 s at 58 °C, and 30 s at 72 °C, and was terminated at 72 °C for 7 min. The PCR product was purified and then directly sequenced using the Sanger sequencing method by U2Bio sequencing service (U2Bio Co., Ltd, South Korea). The phylogenetic tree based on a 992 bp nucleotide sequence of the *omp*A gene fragment was generated by MEGA11 version 11.0.13 using the Neighbor-Joining method with a bootstrap value based on 1000 replicates [13]. The sequences were compared with the corresponding nucleotide sequences from other *Chlamydia* species. All the sequences used in this study were retrieved from the GenBank database. The nucleotide identity of *omp*A sequence alignment was calculated using the Sequence Identity and Similarity program (http://imed.med.ucm.es/Tools/sias.html).

## Results

**Fig. 1:**
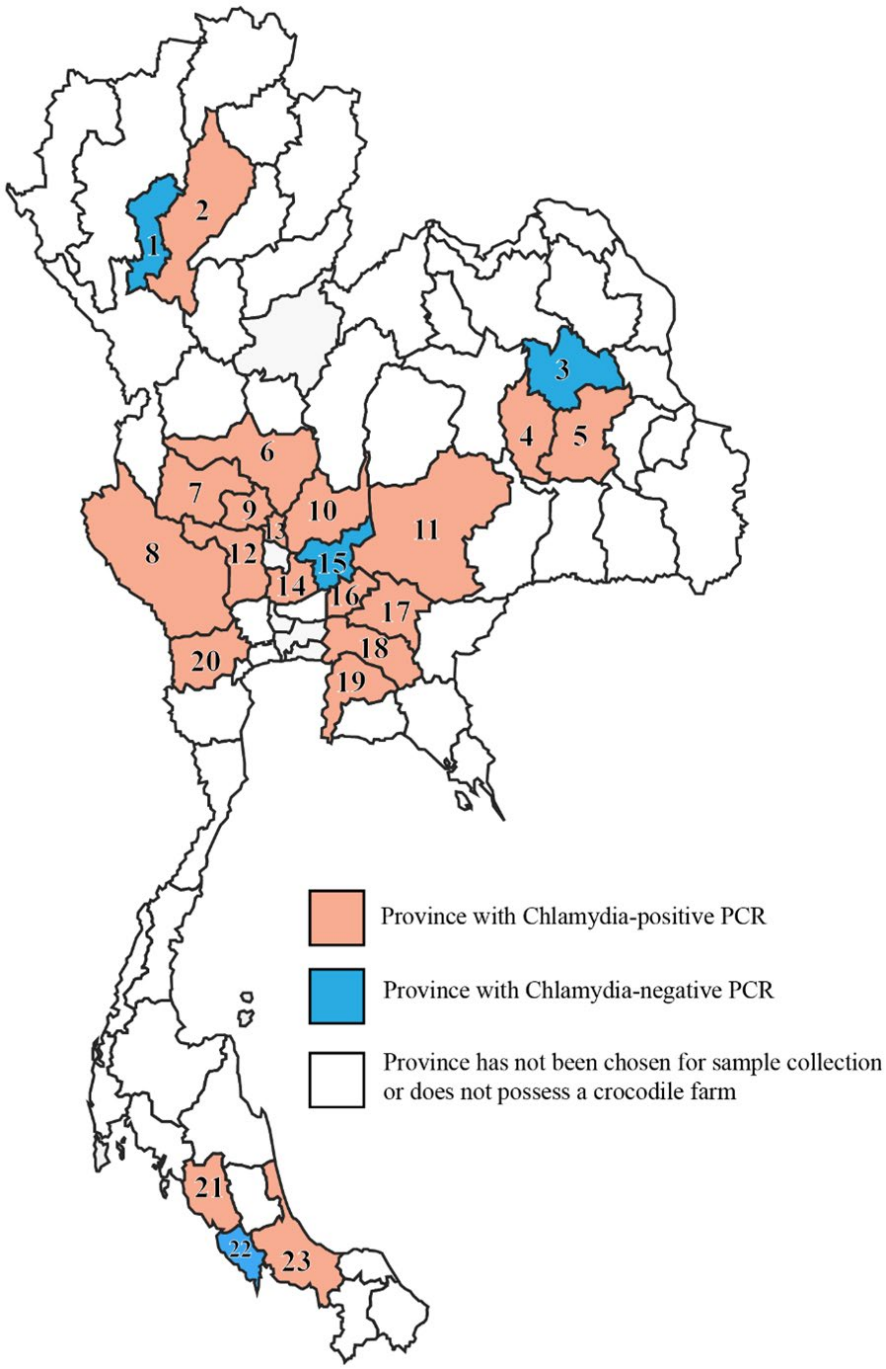
Provinces in Thailand with PCR results positive for Chlamydia sp. See Additional Table S1 for the numbers of farms and crocodiles tested for each province. 1: Lamphun, 2: Lampang, 3: Kalasin, 4: Mahasarakham, 5: Roi Et, 6: Nakhon Sawan, 7: Uthaithani, 8: Kanchanaburi, 9: Chainat, 10: Lopburi, 11: Nakhon Ratchasima, 12: Suphanburi, 13: Singburi, 14: Phra Nakhon Si Ayutthaya, 15: Saraburi, 16: Nakhon Nayok, 17: Prachinburi, 18: Chachoengsao, 19: Chonburi, 20: Petchaburi, 21: Trang, 22: Satun, and 23: Songkhla

*Chlamydia* sp. was detected in 189 samples from 26 farms, representing a prevalence of 65% of all farms. Positive samples were found in all regions of Thailand (Table 1 and Fig. 1), with the highest prevalence detected in the Western region (75%). *Chlamydia* sp. was detected in different swab sites (Table 2), with the highest rate in the cloaca (98.9%), followed by the pharynx (57.1%) and conjunctiva (51.5%).

**Table 1:** Detection rate and prevalence of *Chlamydia* sp. on crocodile farms in Thailand in 2019

Region	Sample*	Farm*	Prevalence on farm level
North	5/25	1/2	50%
Central	77/175	11/15	73%
East	37/84	4/6	66%
North-east	22/80	4/7	57%
West	25/50	3/4	75%
South	23/70	3/6	50%
Total	189/486	26/40	65%

^*^ Data is shown as the number of *Chlamydia* sp. detected samples or farms per total number of analyzed samples or farms, respectively

**Table 2:** Detection rate of *Chlamydia* sp. from conjunctival, pharyngeal, and cloacal swabs

Region	Total positive sample	Swab site		
		Conjunctiva	Pharynx	Cloaca
North	5	3/5	0/0	4/5
Central	77	28/71	23/51	52/73
East	37	12/23	16/31	37/37
North-East	22	13/22	6/10	22/22
West	25	17/25	13/19	25/25
South	23	14/23	14/15	23/23
Total	189	87/169 (51.5%)	72/126 (57.1%)	183/185 (98.9%)

**Fig. 2:**
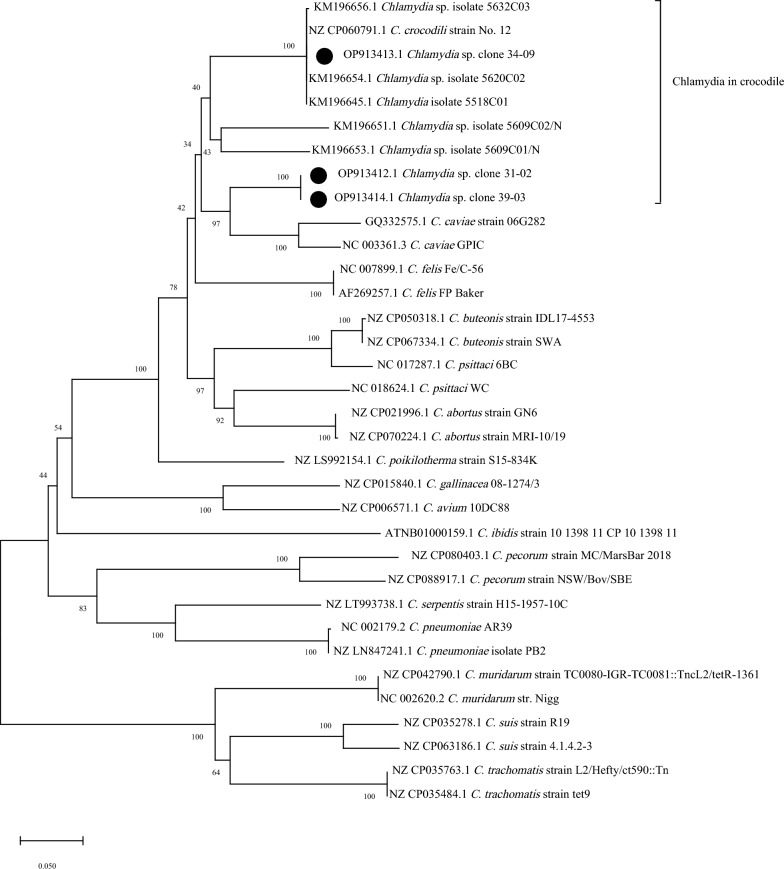
The *omp*A phylogenetic tree. A total of 992 bp nucleotide sequence was used for phylogenetic construction using MEGA11. Numbers show the percentage of times each branch was found in 1000 bootstrap replicates. The tree is drawn to scale, with branch lengths in the same units as those of the evolutionary distances used to infer the phylogenetic tree. The black circle indicates the samples obtained in this study

Although 12 *Chlamydia*-positive samples were sequenced for nearly the full-length of the *omp*A gene, only 3 *Chlamydia*-positive samples were successfully sequenced because of the low DNA quantities of the other samples. The sequenced samples originated from the Eastern (31–02), Northeastern (34–09), and Western region (39–03) and were submitted to GenBank (NCBI) and registered under accession number OP913412.1-OP913414.1. The *omp*A phylogeny demonstrated that sample 34–09 was grouped into the same group as *C. crocodili* (Fig. 2), and this sample exhibited 100% sequence similarity with *C. crocodili* strain No. 12 (accession no. NZ CP060791.1). Samples 31–02, and 39–03 were clustered in a different phylogenetic group. The nucleotide sequences of these samples were 86.99% and 85.28% identical to *Chlamydia caviae* GPIC (accession no. NC 003361.3) and *C. crocodili* strain No. 12 (accession no. NZ CP060791.1), respectively.

Risk factor analyses and odd ratios of various factors for *Chlamydia* sp. infection in crocodiles are presented in Table 3 and Additional file 1. The following factors significantly correlated with *Chlamydia* sp. detection: water source (P = 0.003), reuse of treated wastewater from the treatment pond (P = 0.04), disposal of leftover feed (P = 0.022), water replacement frequency for juvenile crocodiles (P = 0.001), and water replacement following the presence of a deceased crocodile (P = 0.036). *Chlamydia* sp. detection also exhibited a tendency to correlate with the presence of nearby livestock areas (P = 0.056).

**Table 3:** Notable risk factors of *Chlamydia* sp. infection on Siamese crocodile farms (P-value < 0.05)

Factors	Category	Odds ratio	95% CI	P-value
Water source	Natural water with or without treatment		1	0.003
	Tap water	0.096	0.018–0.518	
Reuse treated wastewater from the treatment pond	No		1	0.040
	Yes	4.958	1.009–24.37	
Management of feed leftover	Leave at the pen		1	0.022
	Dispose*	0.01	0.01–0.025	
Water replacement frequency in juveniles	Less than 2 weeks		1	0.001
	More than 2 weeks	12.820	0.015–0.403	
Water replacement following the presence of a deceased crocodile	Yes		1	0.036
	No	8.125	0.917–72 .021	

^*^Force–feeding to the mouth of another crocodile on the farm or toss away from the farming area.

For details of all 26 risk factors analyses, please see Additional file 1

## Discussion

*Chlamydia* sp. causes diseases in a vast variety of vertebrates [6, 14, 15]. In reptiles, this pathogen is commonly reported in crocodilian species, accounting for approximately 57% of all reported cases [14]. *Chlamydia* sp. infection in crocodiles has been reported in several countries in Africa, Thailand, North America, and Australia [2, 6, 8, 15]. However, epidemiological studies seem to be limited, and the information available is mostly from sporadic reports of diagnosed cases.

One study of the prevalence of *Chlamydia* sp. infection, in farmed Siamese crocodiles in the central region of Thailand during 2012–2013, [2] demonstrated different results than our present study (74% and 44%, respectively). The higher detection rate observed in the preceding study could potentially be attributed to the sampling methodology in which specimens were obtained from dead crocodiles that previously had shown clinical manifestations depression and anorexia. This differs from our current investigation, in which samples were collected from live crocodiles. Another study also investigated the prevalence of *Chlamydia* sp. on farms in the Northeastern region of Thailand [3] and reported a prevalence of 48.9%, whereas we found a prevalence of 27.5% (Additional file 2). The observed disparity could potentially arise from variations in the timing of sample collection. Specifically, the previous study collected samples from January to June, whereas we collected samples from July to December. It is noteworthy that the potential impact of the data collection period within a year on pathogen detection has not been investigated yet and should be elucidated in a future study.

The cloacal swab provided the highest detection rate compared with the conjunctiva and pharynx (Table 2), which is in agreement with the results of a previous study [3]. However, this finding contradicts the study of Paungpin et al. [9] in which pharyngeal swabs revealed a 100% *Chlamydia* sp. infection rate. This may be explained by the fact that the samples were collected from severely moribund or dead crocodiles. In our current study, samples were randomly collected from animals with various degrees of clinical signs, which may have led to a lower detection rate of pharyngeal and conjunctival swabs.

Based on the results of our study, feces, and cloacal secretion can play an important role in horizontal disease transmission among crocodiles in affected ponds. Furthermore, cloacal swabs can provide the highest sensitivity and are recommended as the preferred sample collection technique in animals with mild to moderate clinical signs and on farms with low morbidity rates. Additionally, this technique is low-invasive, and does not damage the skin that may lead to poor leather quality. Cloacal swabs also have the following advantages: ease of animal handling, good access to collection sites, time-efficient, and a low risk of traumatic injury to animals and researchers compared with other swab sites, including conjunctiva, and pharynx. Performing pharyngeal swabs is more difficult and presents the highest risk of occupational hazard for researchers and curators while swabbing restrained animals, especially adult crocodiles. Hence, we suggest the cloaca as the sample collection site for *Chlamydia* sp. detection in live crocodiles.

Sequencing and phylogenetic analysis of *omp*A revealed that sample 34–09 was 100% identical to *C. crocodili* strain No. 12, whereas samples 31–02, and 39–03 were grouped together in another cluster. A previous report demonstrated that based on *omp*A gene characterization, *Chlamydia crocodili* detected in Siamese crocodiles in the Central region of Thailand may be divided into three different genotypes [2]. Thus, *Chlamydia crocodili* detected in our study may contain at least two genotypes. However, these genotypes should be further investigated by whole genome sequencing.

The previous study demonstrated variations in *Chlamydia* sp. infection risks among crocodiles emanating from different companies. Many crocodile farms do not breed the crocodile within their farms but receive growing crocodile from the breeder farms. These may pose the risk for disease outbreak if the breeder farm has no measure for disease prevention before sending the crocodile to the growing farm or if the growing farm has no quarantine measure. However, our study analyzed the risks of both farms that had breeding stock within their farm and farms that received growing crocodile from the breeder and found no correlation to the detection of the *Chlaymydia* sp. (Additional file 1). All risk factors we identified in the present study to be associated with the finding of *Chlamydiae* sp. were environmental and management-related factors. Thus, further studies of the detection of the pathogen in the environment of the farm are required for confirmation of the source of the *Chlamydia* sp.

Many farmers feed crocodiles with carcasses of other dead livestock, which poses the risk of *Chlamydia* sp. outbreaks among crocodiles due to infected livestock carcasses. In our study, the presence of livestock in the vicinity of crocodile farms did not pose a significant risk factor but only a trend toward significance (P = 0.056). In addition, crocodiles in Thailand are usually farmed in open areas, which enables potential disease transmission between free-ranging animals, particularly *Chlamydia psittaci* from wild birds, to captive crocodiles. This spillover phenomenon have occurred and caused major losses in equine production and posed a risk to human health [4, 16–18]. Wild pigeons are distributed across all regions of Thailand and are often carriers of *Chlamydia* sp., which can be found in both respiratory and gastrointestinal organs of even asymptomatic pigeons [19]. In birds, *C. psittaci* occurs as an endemic infection affecting 1–5% of the bird population globally, with recovered birds acting as lifelong asymptomatic shedding carriers [4, 15]. Because of the abovementioned reasons, interactions between wild birds, and crocodiles are considered a potential risk factor for *Chlamydia* sp. infection.

Other characteristics of the pond environment, including water depth and the presence of shade, were not identified as risk factors for *Chlamydia* sp. infection. This finding is in accordance with the study by Inchuai et al. [3]. Although water usage and drainage on farms were not found to be risk factors in a previous report [3], using a natural water source was a significant risk factor in our study. The data on the water treatment protocols used on the farm before using in the farm were collected. However, these protocols, including the types of chemicals or natural substances used and the duration of treatment, varied highly among farms and could not be grouped for further analysis. The frequency of water replacement was a risk factor in a previous study by Inchuai et al. [3]. This frequency affects crocodile health because water replacement reduces waste and ammonia levels [20, 21]. In this study, it was observed that extending the duration between water replacements to more than two weeks significantly elevated the risk of *Chlamydia* sp. infections in juvenile crocodiles (Table 3).

During the survey, when sick, and dead crocodiles were found on the farms, farmers treated the animals with drugs (of unknown type and amount) and increased the frequency of water replacements according to the advice of the companies that provide crocodiles and the Fisheries District Officers. This may result in drug contamination in the environment, and the antimicrobial resistance risk. Our study revealed that the reuse of treated wastewater from the water treatment pond is a risk factor for *Chlamydia* sp. detection. However, data regarding the water treatment protocol were not collected. Nevertheless, reusing wastewater may result in reinfections on the farm or environmental contamination.

A notable characteristic of *Chlamydia* sp. is its persistence across different temperatures and climates [22]. In Thailand, *C. psittaci* can thrive with effective infectivity at the high temperature of 56 ℃ for 72 h. Moreover, *Chlamydia* sp. could be retrieved from nature without prior, isolation and cultivation of the natural host cells [21, 23]. Such resilience of *Chlamydia* sp. is in accordance with the environmental risk factors identified in the present study: using natural water sources, not disposing of leftover feed, and not replacing water after the death of a crocodile. Mitigating *Chlamydia* sp. infections on crocodile farms can potentially be achieved by implementing biosecurity measures and environmental sample testing.

## Conclusions

The present study demonstrated that *Chlamydia* sp. infection in Siamese crocodile farming in Thailand during July to December 2019 was 65%. For *Chlamydia* sp. detection, cloacal swabs were superior to conjunctival and pharyngeal swabs due to their higher sensitivity in detecting *Chlamydia* sp., as well as their lower invasiveness. Phylogenetic analysis revealed two potential *Chlamydia* sp. genotypes. The following risk factors associated with *Chlamydia* sp. detection were determined: the use of natural water sources, reuse of treated wastewater from the water treatment pond, no disposal of leftover feed, low frequency of water replacement in juvenile crocodiles, and no water replacement after the death of a crocodile. These data provide useful information for establishing proper guidelines and concepts of disease management and control to prevent disease transmission on crocodile farms. Nevertheless, more detailed studies regarding environmental *Chlamydia* sp. contamination and biosecurity are needed. Moreover, studies regarding antibiotic susceptibility and pathogen resistance are required to establish efficient treatment regimens.

### Supplementary Information

**Additional file 1: **All 26 risk factors of *Chlamydia* sp. infection on Siamese crocodile farms analyzed in the present study.

**Additional file 2: **The number of* Chlamydia*-positive PCR samples collected from crocodiles on farms in different regions and provinces.

## Data Availability

The datasets supporting the conclusions of this article are included within the article and its additional files. Data have not been published previously.
