# Supplementary material for: Epidemiology of Chlamydia sp. infection in farmed Siamese crocodiles (Crocodylus siamensis) in Thailand
Source: Acta Vet Scand. 2023 Nov 27;65:50. doi: 10.1186/s13028-023-00713-x (PMC10680321; doi:10.1186/s13028-023-00713-x)
Supplement: Supplementary file 1 — Additional file 1: All 26 risk factors of Chlamydia sp. infection on Siamese crocodile farms analyzed in the present study. [file 13028_2023_713_MOESM1_ESM.pdf]

Additional file 1 All 26 risk factors of *Chlamydia* sp. infection on Siamese crocodile farms analyzed in the present study.

Factors	Category	Odds ratio	95% CI	P-value
Farm primary objectives	Skin		1	0.84
	Meat	0.667	0.111–3.99	
	Breeding	1.111	0.2–6.181	
	Breeding, and skin or meat	0.333	0.017–6.654	
Crocodile sources	Breeding inside the farm		1	0.723
	From other farms	0.778	0.193–3.13	
Crocodiles raised in the farm	Juvenile		1	0.972
	Adult	0.975	0.242–3.931	
Aquatic livestock on the farm	Yes		1	0.748
	No	1.27	0.295–5.461	
Avian livestock in the farm	Yes		1	0.12
	No	0.274		
Pen floor	Concrete		1	0.778
	Soil	1.8	0.340–9.538	
	Mixed	1.8	0.124–26.195	
The ratio of dry and wet area	<50% dry area		1	0.066
	50% dry area	0.125	0.008–1.998	
	>50% dry area	0.958	0.090–10.235	
Shade	No		1	0.389
	Yes	0.383	0.041–3.613	

Water source	Natural water with or without treatment		1	0.003[a]
	Tap water	0.096	0.018–0.518	
Nearby livestock areas	No		1	0.056
	Yes	4.407	0.912–21.301	
Water reservoir	No		1	0.817
	Yes	1.2	0.257–5.612	
Reuse treated wastewater from the treatment pond	No		1	0.04[a]
	Yes	4.958	1.009–24.37	
Water quality checking	Yes		1	0.274
	No	2.311	0.505–10.573	
Feed source	Industrial slaughterhouse		1	0.477
	Local market	1.964	0.482–7.995	
Food storage	No		1	0.439
	Yes	0.557	0.125–2.481	
Feed additives	Antibiotics		1	0.797
	Vitamins	2.187	0.214–22.337	
	None	1.167	0.236–5.760	
Management of feed leftover	Leave at the pen		1	0.022[a]
	Dispose*	0.01	0.01–0.025	
Pond cleaning practice	Change water only		1	0.208

	Change water and scrub	2.125	0.252–17.927	
	Change water, scrub, and apply disinfectants	10.00	0.584–171.20	
Water replacement frequency in juveniles	Less than 2 weeks		1	0.001[a]
	More than 2 weeks	12.820	0.015–0.403	
Pond preparation	Adjusting pH		1	0.234
	Not adjusting pH	0.417	0.097–1.792	
	Disinfectant use		1	0.49
	No disinfectant use	1.714	0.369–7.974	
	Sundry (>24 h)		1	0.861
	None	1.143	0.257–5.087	
Routine health monitoring	Yes		1	0.465
	No	2.8	0.16–49.103	
Previous health problems	Yes		1	0.439
	No	1.796	0.403–8.00	
Depressed signs**	Detected		1	0.235
	Not detected	2.266667	0.539–.526	
Vertebral deformities	Detected		1	0.321
	Not detected	0.3	0.317–2.434	
Dead crocodile management	Animal or human consumption		1	0.57
	Others	1.2	0.257–5.612	

Water replacement following the presence of a deceased crocodile	Yes		1	0.036[a]
	No	8.125	0.917–72.021	

*Force–feeding to the mouth of another crocodile on the farm or toss away from the farming area

**Signs of depression are anorexia, withdrawal from the group, and hiding

[a] Considered significantly correlated to *Chlamydia* sp. detection (P < 0.05)
