# Supplementary material for: Epidemiology of Chlamydia sp. infection in farmed Siamese crocodiles (Crocodylus siamensis) in Thailand
Source: Acta Vet Scand. 2023 Nov 27;65:50. doi: 10.1186/s13028-023-00713-x (PMC10680321; doi:10.1186/s13028-023-00713-x)
Supplement: Supplementary file 2 — Additional file 2: The number of Chlamydia-positive PCR samples collected from crocodiles on farms in different regions and provinces. [file 13028_2023_713_MOESM2_ESM.pdf]

Additional Table 2 The amount of Chlamydia-positive PCR samples from crocodile samples collected from crocodile farms in different regions and provinces.

Region	Province	Positive/total sample in the province (%)	Positive/total sample in the region (%)
North	Lampang	5/11 (45.4%)	5/25 (20.0%)
	Lamphun	0/14 (0%)	
Central	Suphanburi	13/29 (44.8%)	77/175 (44.0%)
	Singburi	13/14 (92.9%)	
	Lopburi	20/28 (71.4%)	
	Nakhon Nayok	2/14 (14.3%)	
	Saraburi	0/24 (0%)	
	Uthaithani	1/14 (7.1%)	
	Nakhon Sawan	8/23 (34.8%)	
	Chainat	5/14 (35.7%)	
	Phra Nakhon Si Autthaya	15/15 (100%)	
East	Chachoengsao	10/28 (35.7%)	37/84 (44.0%)
	Chonburi	13/42 (30.9%)	
	Prachinburi	14/14 (100%)	
Northeast	Nakhon Ratchasima	4/14 (28.6%)	22/80 (27.5%)
	Roi Et	8/39 (20.5%)	
	Mahasarakham	10/14 (71.4%)	
	Kalasin	0/13 (0%)	
West	Kanchanaburi	6/28 (21.4%)	25/50 (50%)
	Ratchaburi	19/22 (86.4%)	
South	Songkhla	18/44 (40.9%)	23/72 (31.9%)
	Satun	0/14 (0%)	
	Trang	5/14 (35.7%)	
Total			189/486 (38.9%)
